# Validation of the Brasil-Nutri software for 24-hour dietary recall using recovery biomarkers

**DOI:** 10.1590/1980-549720260054

**Published:** 2026-10-19

**Authors:** Jéssica Levy, Letícia Lopes Takarabe, Eduardo De Carli, Sandra Patricia Crispim, Rosângela Alves Pereira, Edna Massae Yokoo, Karina Pfrimer, Daniela Dalpubel Campanari, Meire Gallo Rosenburg, Paulo Lotufo, Isabela Bensenor, Roseli Sichieri, Jeanne Hubertha Maria de Vries, Dirce Maria Lobo Marchioni

**Affiliations:** I Universidade de São Paulo, Faculty of Public Health Department of Nutrition – São Paulo (SP), Brazil.; II Universidade Federal do Paraná, Department of Nutrition– Curitiba (PR), Brazil.; III Universidade Federal do Rio de Janeiro, Department of Social and Applied Nutrition – Rio de Janeiro (RJ), Brazil.; IV Universidade Federal Fluminense, Institute of Collective Health – Niterói (RJ), Brazil.; V Universidade de Ribeirão Preto, Department of Nutrition – Ribeirão Preto (SP), Brazil.; VI Universidade de São Paulo, Faculty of Medicine of Ribeirão Preto – Ribeirão Preto (SP), Brazil.; VII Universidade de São Paulo, University Hospital, Center for Clinical and Epidemiological Research – São Paulo (SP), Brazil.; VIII Universidade Estadual do Rio de Janeiro, Institute of Social Medicine, Department of Epidemiology – Rio de Janeiro (RJ), Brazil.; IX Wageningen University & Research, Human Nutrition & Health – Wageningen, The Netherlands.

**Keywords:** Software, Diet surveys, Energy expenditure, Biomarker, Validation study

## Abstract

**Objective:**

To evaluate measurement errors in duplicate 24-hour dietary recalls, guided by the Brasil-Nutri software, using recovery biomarkers for energy, protein, sodium, and potassium in a subsample of participants from the Brazilian Longitudinal Study of Adult Health (ELSA-Brasil).

**Methods:**

Apparently healthy men and women with stable body weight were included in the study. Two non-consecutive 24-hour dietary recalls, guided by the Brasil-Nutri software, were compared with recovery biomarkers of protein, potassium, and sodium obtained from two 24-hour urine samples. Total energy expenditure was assessed using the doubly labeled water method.

**Results:**

Eighty individuals, aged 43 to 72 years, participated in the study. Self-reported energy intake (2,146 kcal/day) was lower than the total energy expenditure estimated by the doubly labeled water method (2,526 kcal/day), with a mean difference of 380 kcal/day. Self-reported protein intake (94.9 g/day) was higher than the biomarker estimate (87.6 g/day). For sodium, mean self-reported intake was 2.9 g/day compared with 4.7 g/day estimated by the biomarker, while potassium intake was 2.9 g/day *versus* 3.0 g/day, respectively.

**Conclusion:**

The Brasil-Nutri software showed acceptable performance for estimating population mean intake of some nutrients; however, important measurement errors were observed, particularly for sodium intake. In addition, correlations with recovery biomarkers were low to moderate, indicating limitations in individual-level agreement.

## INTRODUCTION

Food intake is commonly assessed using self-report-based dietary methods that are subject to random and systematic errors^1^. Understanding these errors is essential to interpret the results of studies in nutritional epidemiology or to correct for them. To evaluate the validity of dietary assessment methods, their estimates are compared with those obtained from reference methods with independent measurement errors^2,3^. Advances in dietary assessment technologies have reduced the time and costs required for data collection, coding, and data analysis. However, it is necessary to also validate these adapted methods^4-9^.

Brasil-Nutri is a computerized 24-hour dietary recall system developed to support national dietary surveys in Brazil, including the National Food Survey^10-12^. The software includes an extensive food database based on previous national dietary surveys and epidemiological studies^13^.

Recovery biomarkers provide objective measures of dietary intake and are widely used in validation studies because their measurement errors are largely independent of self-reported methods^1^. These biomarkers include 24-hour urinary excretion of nitrogen, sodium, and potassium, which can be used to estimate dietary protein and mineral intake^14^.

The aim of this study was to evaluate measurement errors in duplicate 24-hour dietary recalls collected using the Brasil-Nutri software by comparison with recovery biomarkers in a subsample of ELSA-Brasil participants. Energy intake was evaluated using the doubly labeled water (DLW) method, whereas protein, sodium, and potassium intakes were assessed using urinary recovery biomarkers. Performance was evaluated at both the population and individual levels.

## METHODS

### Study sample

The Longitudinal Study of Adult Health (ELSA-Brasil) is a multicenter cohort investigation comprising 15,105 participants, of both sexes, active and retired workers from six Brazilian states, with the main objective of investigating the incidence and risk factors for chronic non-communicable diseases^15^. The present study was carried out at the ELSA-Brasil research center in São Paulo, located at the Clinical and Epidemiological Research Center of the University Hospital of the University of São Paulo (CPCE/HU/USP).

Between August 2018 and December 2019, 101 adults and older adults of both sexes were recruited. Participant selection and exclusions are described in Figure 1. To validate the estimates of dietary protein, sodium, and potassium collected using the Brasil-Nutri software, 18 individuals were excluded due to missing dietary assessment data and/or incomplete 24-hour urine collection. Additionally, three participants without DLW information were excluded. Thus, the final analytical sample comprised 80 participants.

**Figure 1:**
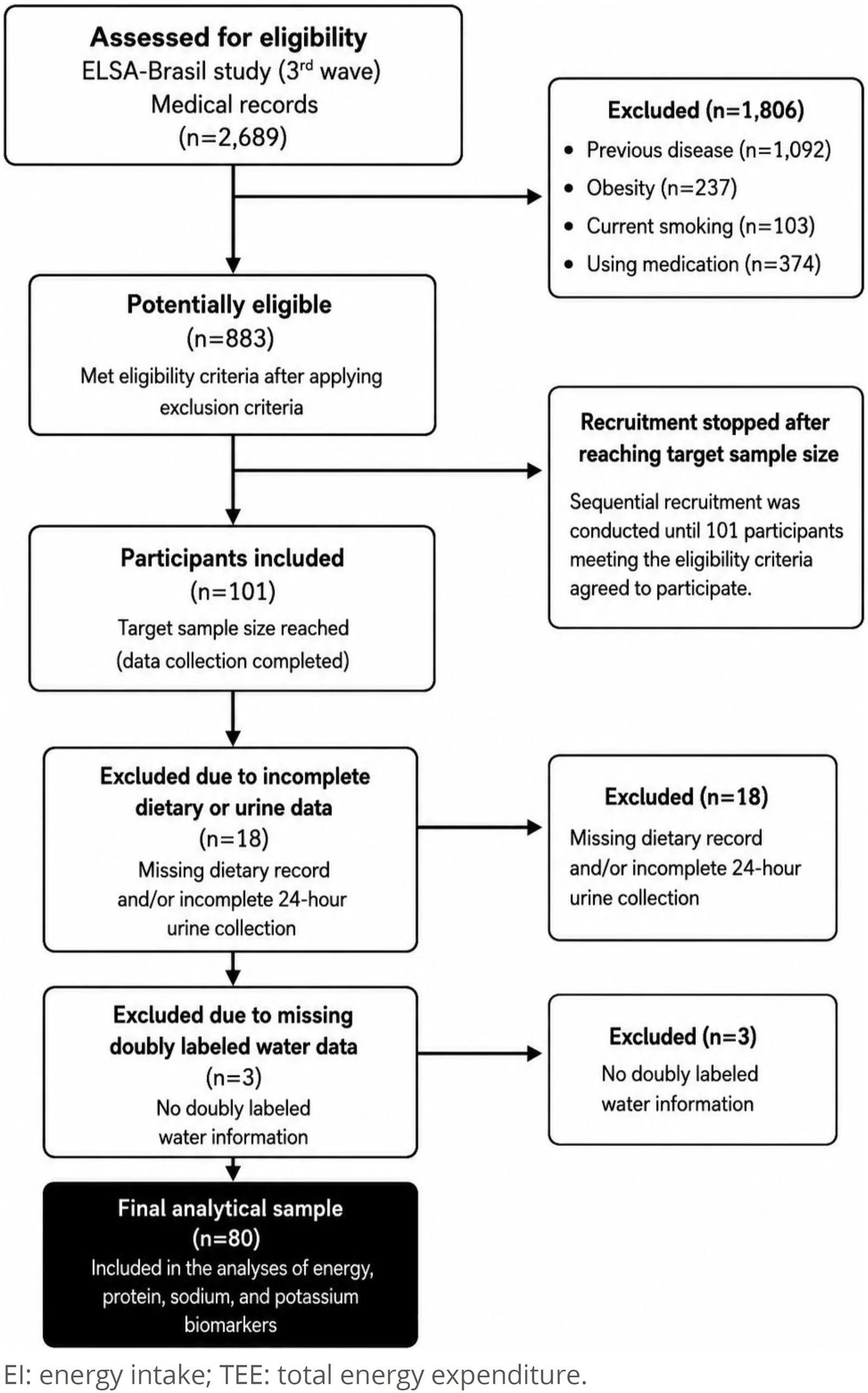
Volunteer recruitment flowchart.

All participants received detailed instructions about the collection protocol and signed an Informed Consent Form, indicating their intention to participate in this study. The study was approved by the Research Ethics Committee of the University Hospital of the University of São Paulo.

### 24-hour dietary recalls guided by the Brasil-Nutri software

Two non-consecutive 24-hour dietary recalls (24hR), with an interval of 3 to 4 weeks between assessments, were collected through the Brasil-Nutri software. The study protocol was designed so that the first 24hR interview was conducted face-to-face at the study center and the second, by telephone. However, for participants who had difficulty completing the telephone interview or presented limitations related to remote contact, the second interview was also conducted face-to-face in order to minimize losses to follow-up. The telephone interview strategy was adopted to evaluate the operational feasibility of remote dietary assessment, similarly to procedures used in large epidemiological studies. In addition, all participants completed an initial face-to-face interview with visual support for portion size estimation and household measures.

The 24hR interview guided by the Brasil-Nutri software begins with questions regarding the habitual use of salt and sugar or sweeteners, nutritional supplements, and adherence to restrictive diet patterns or specific diet therapies. The respondent was then asked to mention all food and beverages consumed 24 hours before, the place and time of consumption, as well as details about each food and beverage, including the method of preparation, type of unit, and the amount of consumption in household measurements or standard units, illustrated by images on the computer screen^16^. Nutrient estimation was based on the Brazilian Food Composition Table (TBCA/USP)^17-19^. Sodium values available in the food composition database include intrinsic sodium, sodium added during food processing, and estimated sodium from culinary preparations.

### Anthropometric and lifestyle data

Each participant completed a questionnaire on socioeconomic characteristics and lifestyle factors. Physical activity was classified according to the International Physical Activity Questionnaire (IPAQ) as light, moderate, or vigorous^20^. Due to the small number of participants in the vigorous category, moderate and vigorous levels were combined. Social class was categorized in accordance with the Criteria for Economic Classification of the Brazilian Association of Research Companies, with class high having ≥45 points; class middle ≥29 and ≤44 points, and class low ≤28 points^21^.

Anthropometric measurements were performed duplicate, with the participants wearing light clothing, without shoes and adornments, and with empty pockets. Height, weight, and waist circumference were measured to the nearest of 0.1 cm, 0.1 kg, and 0.1 cm, respectively, with calibrated equipment at the beginning and end of the study. Average weight and height measurements were used to calculate the body mass index (BMI [kg/m^2^]) and classify the nutritional status of the individuals, following the criteria of the World Health Organization^22^ for adults and the Lipschitz criteria^23^ for older people. Participants were classified as: 1) underweight with BMI<18.5 kg/m^2^ for adults and BMI<22 kg/m^2^ for older people; 2) normal weight (BMI 18.5–24.9 kg/m²) for adults and BMI<27 kg/m^2^ for older people; and 3) overweight with BMI≥25 kg/m^2^ for adults and BMI≥27 kg/m^2^ for older participants. However, as only one participant was underweight, this category was combined with the normal-weight group.

### Recovery biomarkers

Two 24-hour urine samples (24hU) were collected from each participant as part of the study protocol. The first 24hU sample was collected on the same day as the first 24hR assessment. The second 24hU sample was collected after completion of the two 24hR assessments, during a subsequent evaluation period. The average interval between urine collections was six months (minimum of one and maximum of 14 months). For the analyses, the mean of the two urine collections was used as the reference biomarker measure in order to reduce within-person variability and improve estimation of habitual intake.

24hU collections that had a collection interval of less than 22 hours or greater than 26 hours were considered invalid. Total urine collection time was calculated as the interval between the recorded start and end times of collection. Collections with a 24hU volume lower than 500 mL or with total creatinine excretion outside the expected range, <14.4 or >33.6 mg/kg for men and <10.8 or >25.2 mg/kg for women, as suggested by Pereira et al.^24^, were also considered invalid.

The determination of urinary urea was performed by a kinetic method (urease and glutamate dehydrogenase), performed by the Cobas c501^®^ automated analyzer (Roche^®^). Values of total urea excretion in 24h were converted to nitrogen (g/day), assuming that urea is a constant/stable portion (85%) of the total urinary nitrogen^25,26^. Next, protein intake was calculated by the following formula: protein intake = 6.25 * (urinary nitrogen (g/day) + 2), where the +2 represents extra-renal nitrogen losses (stool and skin) in g/day^27^. Urinary potassium concentrations were measured using the ion-selective electrode technique. Dietary potassium intake was calculated by dividing urinary potassium excretion by 0.77. Urinary sodium concentrations were measured using the ion-selective electrode technique. Dietary sodium intake was calculated by dividing urinary sodium excretion by 0.86, as per Freedman et al.^28^.

Total energy expenditure (TEE) over a 2-week period was assessed using the double labeled water (DLW) in 101 participants. The DLW protocol was repeated in 26 participants to evaluate reproducibility and improve the precision of TEE estimates. Doses were prepared with a ratio of 2 g of 10% ^18^O and 0.11 g of 99.8% deuterium oxide (^2^H_2_O) per kilogram of estimated total body water^29^. A baseline urine sample was collected before the participants ingested the DLW dose. After ingestion, a sample was collected every day for 14 days, preferably at the same time at the participants’ homes. On the 7th and 14th days of collection, the samples of the week were delivered to the laboratory, and the samples of the day were collected at the time of the interview at the investigation center. However, only samples of baseline and of days 1, 2, 3, 7, 8, 13, and 14 were analyzed. All samples were refrigerated until delivered for processing. Enrichment of ^2^H and ^18^O isotopes in urine samples was analyzed in triplicate using isotope ratio mass spectrometry (IRMS-GLS: ANCA 20-20, Europe Scientific, UK and ANCA 22-20, Sercon, UK) at the Laboratory of Isotope Ratio Mass Spectrometry, Faculty of Medicine of Ribeirão Preto, University of São Paulo, Brazil. The decay ratio values between ²H and ^18^O ranged from 1.01 to 1.07. Body weight was monitored throughout the DLW measurement period to ensure participants were not losing or gaining weight. Participants were considered weight-stable when the weight difference between days 1 and 14 was <1 kg.

### Statistical analysis

Data referring to socioeconomic, anthropometric, and lifestyle characteristics were described by frequencies, and associations between categorical variables were tested using the chi-square or Fisher's exact test. The values of dietary variables and biomarkers were presented in crude form. Difference (dietary intake minus biomarker), ratio (dietary intake / biomarker) and relative bias {[(ln dietary intake / ln biomarker) × 100] − 100}, representing the percentage of misreporting at the population level, were estimated^28^. Agreement between methods at both the group and individual levels, overall and stratified by sex, was evaluated using Bland–Altman plots^30^. For the other analyses, the values obtained on two non-consecutive days were adjusted for the distribution of intake by intrapersonal variability, using the Multiple Source Method (MSM) software^31^. Correlation coefficients between dietary variables and their respective biomarkers to evaluate whether the method was able to rank individuals according to their intake were calculated using Pearson correlation coefficient, and Fisher's Z transformations were used to calculate 95% confidence intervals of the correlation coefficients. In all analyses, a significance level of 5% was considered, and the statistical treatment of the data was carried out using Stata^®^ software (version 14.0).

To interpret the validity of the dietary assessment method, different aspects of performance were considered. Agreement at the population level was evaluated by comparing mean dietary intake estimates with recovery biomarkers through differences, ratios, and relative bias. The ability to rank individuals according to intake was assessed using correlation coefficients between dietary estimates and biomarkers, with correlations classified as low (<0.30), moderate (0.30–0.50), or strong (>0.50). Agreement at the individual level was additionally evaluated using Bland–Altman plots and limits of agreement.

#### Data availability statement:

The data described in the manuscript can be made available upon request, pending application and approval by the chair of the steering committee for the Brazilian Longitudinal Study of Adult Health (ELSA-Brasil) Brasil-Nutri validation study.

## RESULTS

Among the 80 study participants, 43 were women and 37 were men. Most (86.3%) were aged between 43 and 59 years. More than half had completed a university degree or postgraduate studies (56.2%), 62.5% belonged to the middle socioeconomic class, 55.0% reported a light level of physical activity, and 63.8% were classified as overweight (Table 1).

**Table 1:** Socioeconomic and lifestyle characteristics of a sub-sample from the Longitudinal Study of Adult Health (ELSA-Brasil) used for validation of the Brasil-Nutri software.

		Total (n=80)		Men (n=37)		Women (n=43)		p*
		N	%	n	%	N	%	
Age group								
	Adult	69	86.3	32	86.5	37	86.1	0.955
	Older people	11	13.7	5	13.5	6	13.9	
Education								
	High school or less	35	43.8	19	51.4	16	37.2	0.204
	University degree/Postgraduate	45	56.2	18	48.6	27	62.8	
Social class								
	High	17	21.2	7	18.9	10	23.3	0.787
	Top	50	62.5	23	62.2	27	62.8	
	Low	13	16.2	7	18.9	6	13.9	
Body mass index								
	Normal weight	29	36.2	9	24.3	20	46.5	0.040
	Overweight	51	63.8	28	75.7	23	53.5	
Physical activity								
	Light	44	55.0	19	51.4	25	58.1	0.543
	Moderate to vigorous	36	45.0	18	48.6	18	41.9	

Displayed values are count (frequency)

* p-value relative to the comparison between sexes according to the chi-square test.

Adult: 43 to 59 years old; older people are aged 60 years or more. Social class according to the criteria of the Brazilian Association of Research Companies (ABEP): high is greater than or equal to 45 points; top is greater than or equal to 29 and less than or equal to 44 points; low is less than or equal to 28 points. Body mass index: normal weight is less than 25 kg/m^2^ for adults and less than 27 kg/m^2^ for older people; overweight is greater than or equal to 25 kg/m^2^ for adults and greater than or equal to 27 kg/m^2^ for older people Light physical activity: those who did not perform any physical activity for at least 10 continuous minutes during the week or who performed physical activity but insufficiently to be classified as active for not meeting the recommendations regarding frequency (greater than or equal to five days per week) or duration (greater than or equal to 150 minutes per week). Moderate-to-vigorous physical activity: those who met the recommendations of greater than or equal to three days per week and greater than or equal to 20 minutes per session of vigorous activity; greater than or equal to five days per week and greater than or equal to 30 minutes per session of moderate activity or walking; or greater than or equal to five days per week and greater than or equal to 150 minutes with any added activity.

Table 2 presents dietary intake estimates for energy, protein, sodium, and potassium, their respective recovery biomarkers, and the correlations between the two methods. Mean self-reported energy intake (2,146.1 kcal/day) was lower than TEE estimated by DLW (2,526.5 kcal/day), resulting in a mean difference of 380.3 kcal/day. Relative bias indicated underestimation of energy intake in the total sample (-1.8%), as well as among men (-1.6%) and women (-2.0%).

**Table 2:** Dietary and biomarker energy, protein calculated from nitrogen, sodium, and potassium based on two days of 24-hourR recalls and two 24-hour urine collections or doubly labeled water in the Brasil-Nutri validation study.

Variables		Total (n=80)		Men (n=37)		Women (n=43)	
		Mean	95%CI	Mean	95%CI	Mean	95%CI
Energy							
	Diet (kcal/day)	2,146.1	(1,995.7 to 2,296.5)	2,413.9	(2,179 to 2,648.9)	1,915.6	(1,742.7 to 2,088.6)
	Biomarker (kcal/day)	2,526.5	(2,316.1 to 2,736.9)	2,834.9	(2,489.5 to 3,180.4)	2,261.1	(2,021.8 to 2,500.4)
	Difference (kcal/day)	-380.3	(-617.8 to -142.9)	-421.0	(-809.5 to -32.56)	-345.4	(-649.2 to -41.6)
	Ratio	0.9	(0.8 to 1.0)	0.9	(0.8 to 1.1)	0.9	(0.8 to 1.0)
	Relative bias (%)	-1.8	(-3.0 to -0.6)	-1.6	(-3.5 to 0.3)	-2.0	(-3.7 to -0.4)
	Crude correlation	0.15	(-0.07 to 0.36)	0.15	(-0.19 to 0.45)	-0.02	(-0.32 to 0.28)
Protein							
	Diet (g/day)	94.9	(87.2 to 102.7)	111.5	(100.4 to 122.7)	80.6	(71.5 to 89.8)
	Biomarker (g/day)	87.6	(83.2 to 92.1)	98.1	(91.6 to 104.6)	78.7	(73.8 to 83.5)
	Difference (g/day)	7.3	(0.7 to 13.9)	13.5	(2.6 to 24.3)	1.9	(-6.1 to 10.1)
	Ratio	1.1	(1.0 to 1.1)	1.2	(1.0 to 1.3)	1.0	(0.9 to 1.1)
	Relative bias (%)	0.8	(-0.7 to 2.42)	2.3	(0.1 to 4.5)	-0.4	(-2.7 to 1.8)
	Crude correlation	0.6	(0.39 to 0.69)	0.4	(0.10 to 0.65)	0.5	(0.23 to 0.69)
	Deattenuated correlation^1^	0.6	(0.46 to 0.74)	0.3	(0.02 to 0.59)	0.4	(0.06 to 0.59)
Sodium							
	Diet (g/day)	2.9	(2.7 to 3.3)	3.5	(3.0 to 3.9)	2.6	(2.3 to 2.9)
	Biomarker (g/day)	4.7	(4.4 to 5.0)	5.6	(5.1 to 6.1)	3.9	(3.6 to 4.2)
	Difference (g/day)	-1.7	(-2.1 to -1.3)	-2.1	(-2.8 to -1.4)	-1.4	(-1.7 to -0.9)
	Ratio	0.7	(0.6 to 0.8)	0.7	(0.6 to 0.8)	0.7	(0.6 to 0.8)
	Relative bias (%)	-30.7	(-37.1 to -24.3)	-27.7	(-37.5 to -18.0)	-33.2	(-42.1 to -24.4)
	Crude correlation	0.37	(0.16 to 0.55)	0.12	(-0.21 to 0.43)	0.27	(-0.03 to 0.53)
	Deattenuated Correlation^1^	0.52	(0.33 to 0.66)	0.02	(-0.31 to 0.34)	0.11	(-0.19 to 0.39)
Potassium							
	Diet (g/day)	2.9	(2.7 to 3.1)	3.2	(2.9 to 3.5)	2.6	(2.3 to 2.9)
	Biomarker (g/day)	3.0	(2.9 to 3.2)	3.2	(2.9 to 3.5)	2.9	(2.6 to 3.1)
	Difference (g/day)	-0.2	(-0.4 to 0.1)	-0.0	(-0.3 to 0.3)	-0.3	(-0.6 to 0.1)
	Ratio	0.9	(0.9 to 1.1)	1.0	(0.9 to 1.1)	0.9	(0.8 to 1.1)
	Relative bias (%)	-3.7	(-11.9 to 4.6)	-0.1	(-10.6 to 10.3)	-6.7	(-19.6 to 6.1)
	Crude correlation	0.28	(0.06 to 0.47)	0.53	(0.24 to 0.73)	0.01	(-0.29 to 0.31)
	Deattenuated Correlation^1^	0.33	(0.12 to 0.51)	0.34	(0.02 to 0.60)	0.19	(-0.11 to 0.47)

Displayed values are means and 95% confidence intervals (95%CI); Difference: dietary intake – biomarker; Ratio: dietary intake divided by biomarker; Relative bias: {[(ln dietary intake / ln biomarker) × 100] − 100}.

* Dietary and biomarker measurements were obtained from 24-hour recalls and 24-hour urine collections, respectively.

1 Correlation with removal of intrapersonal variability by the Multiple Source Method (MSM).

Mean self-reported protein intake (94.9 g/day) was higher than the biomarker estimate (87.6 g/day), corresponding to a mean difference of 7.3 g/day. Among men, mean self-reported protein intake was 111.5 g/day compared with 98.1 g/day estimated by the biomarker, whereas among women the corresponding values were 80.6 g/day and 78.7 g/day. The correlation coefficient between protein intake and its biomarker was 0.60 (95%CI 0.39–0.69), remaining unchanged after adjustment for within-person variability (r = 0.60; 95%CI 0.46–0.74). For sodium, mean self-reported intake was 2.9 g/day, whereas the biomarker estimate was 4.7 g/day, resulting in a mean difference of 1.7 g/day. For potassium, mean self-reported intake was 2.9 g/day compared with 3.0 g/day estimated by the biomarker, corresponding to a mean difference of 0.2 g/day.

Table 3 presents the mean differences between dietary intake estimates and recovery biomarkers according to socioeconomic, anthropometric, and lifestyle characteristics. No statistically significant differences were observed across the evaluated groups. Nevertheless, underestimation was observed for energy, sodium, and potassium intake in most subgroups. For energy intake, greater underestimation was observed among older adults, overweight participants, and those reporting moderate-to-vigorous physical activity. A distinct pattern was observed across socioeconomic groups, with overreporting among participants in the lowest socioeconomic class and underreporting among those in the middle and highest classes. For sodium and potassium, differences between dietary estimates and biomarkers were relatively consistent across subgroups, although larger discrepancies were observed among older adults. In contrast, protein intake tended to be overestimated among adults, participants from the lowest socioeconomic class, overweight individuals, and those reporting light physical activity.

**Table 3:** Means and 95% confidence intervals of the difference between dietary and biomarker estimates for energy, protein, sodium, and potassium in the Brasil-Nutri software validation study.

Characteristics		Energy (kcal) difference		Protein (g) difference		Sodium (g) difference		Potassium (g) difference	
Age group									
	Adult	-321.3	(-586.9 to -55.8)	7.5	(0.0 to 14.9)	-1.7	(-2.0 to -1.2)	-0.1	(-0.3 to 0.2)
	Older people	-750.7	(-1,229.1 to -272.4)	6.1	(-8.4 to 20.7)	-2.2	(-3.2 to -0.8)	-0.6	(-1.4 to 0.2)
Education									
	High school or less	-488.4	(-882.4 to -4.3)	6.9	(-2.8 to 16.7)	-1.9	(-2.6 to -1.2)	-0.2	(-0.5 to 0.2)
	University degree/Postgraduate	-327.5	(-596.7 to -58.3)	7.6	(-1.8 to 16.9)	-1.6	(-2.0 to -1.1)	-0.1	(-0.5 to 0.2)
Social class									
	High	-493.8	(-1004.6 to 16.8)	0.8	(-5.6 to 7.2)	-1.5	(-2.2 to -0.9)	-0.6	(-1.0 to -0.1)
	Top	-449.6	(-760.2 to -139.1)	8.8	(-1.1 to 18.7)	-1.7	(-2.2 to -1.2)	-0.1	(-0.4 to 0.2)
	Low	34.4	(-592.1 to 661.0)	9.9	(-5.0 to 24.8)	-1.8	(-3.0 to -0.6)	0.1	(-0.5 to 0.7)
Body mass index									
	Normal weight	-185.0	(-429.4 to 59.3)	1.9	(-10.7 to 14.7)	-1.5	(-1.9 to -0.9)	-0.5	(-0.9 to 0)
	Overweight	-491.5	(-839.6 to -143.3)	10.3	(2.6 to 17.9)	-1.8	(-2.4 to -1.3)	0.0	(-0.2 to 0.3)
Physical activity									
	Light	-276.6	(-547.2 to -6.0)	8.6	(-0.9 to 18.1)	-1.6	(-2.1 to -1.1)	-0.2	(-0.5 to 0.2)
	Moderate to vigorous	-507.2	(-930.5 to -83.9)	5.6	(-3.9 to 15.1)	-1.8	(-2.4 to -1.3)	-0.1	(-0.5 to 0.2)

* Negative values indicate underestimation.

Adult: 43 to 59 years old; older people are aged 60 years or more. Social class according to the criteria of the Brazilian Association of Research Companies (ABEP): high is greater than or equal to 45 points; top is greater than or equal to 29 and less than or equal to 44 points; low is less than or equal to 28 points. Body mass index: normal weight is less than 25 kg/m2 for adults and less than 27 kg/m2 for older people; overweight is greater than or equal to 25 kg/m2 for adults and greater than or equal to 27 kg/m2 for older people Light physical activity: those who did not perform any physical activity for at least 10 continuous minutes during the week or who performed physical activity but insufficiently to be classified as active for not meeting the recommendations regarding frequency (greater than or equal to five days per week) or duration (greater than or equal to 150 minutes per week). Moderate-to-vigorous physical activity: those who met the recommendations of greater than or equal to three days per week and greater than or equal to 20 minutes per session of vigorous activity; greater than or equal to five days per week and greater than or equal to 30 minutes per session of moderate activity or walking; or greater than or equal to five days per week and greater than or equal to 150 minutes with any added activity.

Agreement analyses based on Bland–Altman plots demonstrated substantial variability between dietary estimates and recovery biomarkers at the individual level, particularly for energy and sodium intake (Supplementary Figure 1). Potassium showed comparatively narrower limits of agreement, whereas sodium exhibited the greatest dispersion of differences between methods.

## DISCUSSION

This study evaluated measurement errors in 24-hour dietary recalls guided by the Brasil-Nutri software using recovery biomarkers in participants from the ELSA-Brasil study. At the population level, the performance of the method varied according to the nutrient evaluated, with overestimation for protein and underestimation for sodium and potassium when compared with recovery biomarkers. In addition, low to moderate correlations were observed between dietary estimates and biomarkers, particularly for energy intake, indicating limited agreement at the individual level. These findings suggest that, although the software may provide reasonable estimates for some nutrients at the population level, important measurement errors remain and should be carefully considered when interpreting the results.

An increasing number of studies have proposed technological innovations for collecting 24hR, including the use, for example, of smartphone applications and digital images, in order to overcome the logistical challenges in epidemiological studies^5-9,32,33^. Previous validation studies of online dietary assessment tools such as Oxford WebQ and MyFood24 have also reported measurement errors when compared with recovery biomarkers, including overestimation of some nutrients^32,33^. Other studies that compared the estimates of protein, sodium, and potassium obtained by 24hR with urinary biomarkers described a tendency to underestimate the intake of the three nutrients^34-36^. The present study showed an overestimation for protein (94.9 g *versus* 87.6 g) and underestimation for sodium (2.9 g *versus* 4.7 g) and potassium (2.9 g *versus* 3.0 g). In contrast to the underestimation commonly described for dietary intake assessment methods, protein intake was slightly overestimated in the present study. One possible explanation is that protein-rich foods are generally easier to identify and quantify during dietary recalls than nutrients such as sodium, which depend heavily on food preparation practices and discretionary salt use. In addition, the relatively high educational level of the study population may have contributed to more accurate reporting of main meals and protein-containing foods.

Potassium showed comparatively smaller differences and narrower limits of agreement than sodium. This finding may be related to the fact that potassium intake is largely derived from minimally processed foods such as fruits, vegetables, and legumes, which are generally easier to report and less influenced by discretionary preparation practices.

A North American study that used recovery biomarkers as the reference method and included only women reported a deattenuated correlation coefficient of 0.54 for energy-adjusted protein intake assessed using the Automated Self-Administered 24-Hour Dietary Recall (ASA24)^37^, which is comparable to the correlation observed in the present study (r=0.60)^37^. The correlation coefficients observed herein were comparable to those reported in previous validation studies using urinary biomarkers^38^. For potassium, deattenuated correlations were 0.34 for men and 0.19 for women. For sodium, the corresponding values were 0.02 and 0.11, respectively. Although low to moderate, these correlations indicate that the dietary recalls were able to rank individuals according to intake, which is an important characteristic for epidemiological studies.

The substantial underestimation observed for sodium intake represents one of the main findings of this study and highlights an important limitation of the method. Estimating sodium intake through self-reported dietary assessment remains one of the greatest challenges in dietary assessment due to difficulties in quantifying discretionary salt added during food preparation and consumption, as well as the variability of sodium content in processed foods. In addition, sodium intake presents considerable intraindividual variability, which may further reduce agreement between dietary recalls and recovery biomarkers. Body weight has consistently been identified as a predictor of measurement bias in self-reported dietary assessment methods, with a higher degree of underreporting, especially of energy, associated with overweight and obesity^39-45^. Mercado et al.^38^ described a greater underreporting of sodium intake in 24hR collected by the traditional method among overweight and obese individuals. In the present study, sodium estimates obtained by the 24hR compared with its biomarker were underestimated by 1.7 g for the total population and by 2.1 g and 1.4 g for men and women, respectively. Furthermore, underestimation was greater among overweight participants compared with normal-weight participants for this nutrient specifically (1.8 g versus 1.5 g). However, it is important to highlight that individuals with obesity were excluded from the present study as a deliberate methodological strategy to minimize factors previously associated with systematic misreporting in dietary assessment studies. Previous studies comparing dietary recalls with urinary biomarkers have also demonstrated low agreement and substantial underestimation for sodium intake at the individual level^46-49^.

Comparisons with studies involving obese populations should therefore be interpreted with caution; they were included herein primarily to contextualize patterns of dietary misreporting described in the literature. The applicability of the present findings to individuals with obesity remains uncertain and should be further investigated in future validation studies that include more heterogeneous populations.

Another important aspect is that estimating discretionary salt intake remains inherently challenging in dietary surveys, even when specific questions regarding added salt are included. In the Brasil-Nutri software, additional questions related to food preparation and seasoning, including salt addition, were systematically applied to all reported food items. Nevertheless, participants may fail to report salt added to salads and home-prepared foods. These factors may have contributed to the substantial underestimation observed for sodium intake in the present study.

Freedman et al. pooled data from several validation studies and reported energy underreporting ranging from 6% to 28%, depending on the study population and sex^28^. In studies with the ASA 24, the estimated energy intake was lower than the TEE for both men and women^50,51^. In the present study, the underreporting of energy intake with two 24hR using the Brasil-Nutri software was 1.6% for men and 2.0% for women. The underestimation observed for energy intake may be partially explained by omissions of foods perceived as unhealthy, difficulties in recalling snacks and foods consumed outside the home, and challenges in estimating portion sizes. In addition, energy intake is particularly susceptible to social desirability bias and selective underreporting, even among apparently healthy individuals.

The study has several strengths, including the use of strict participant selection criteria designed to minimize factors associated with dietary misreporting, such as chronic diseases, use of drugs/supplements, or with physiological conditions that could compromise the validity of biomarkers or the reporting of consumption; the use of recovery biomarkers for protein, sodium, and potassium validation; and rigorous quality control procedures throughout urine collection, storage, and laboratory analysis. Although the selected sample limits external validity, the inclusion of apparently healthy individuals without obesity or chronic diseases was a deliberate methodological strategy to minimize factors associated with systematic misreporting already described in the literature. This approach allowed evaluation of the software performance under more controlled conditions before broader application in more heterogeneous populations. Therefore, the findings should not be directly extrapolated to the Brazilian population as a whole. Another limitation is that the second biomarker assessment was performed during a later evaluation period, with an average interval of six months between urine collections. In addition, portion-size estimation during telephone interviews was not supported by on-screen food images.

The Brasil-Nutri software demonstrated acceptable performance for estimating mean population intake of some nutrients; however, important measurement errors remained, particularly for sodium intake. Correlations with recovery biomarkers were low to moderate, suggesting limitations in individual-level agreement. These findings support the use of the method with caution in population studies, while highlighting the need for further refinement, especially for sodium estimation.
