# Supplementary material for: Validation of the Brasil-Nutri software for 24-hour dietary recall using recovery biomarkers
Source: Rev Bras Epidemiol. 2026 Oct 19;29:e260054. doi: 10.1590/1980-549720260054 (PMC13679881; doi:10.1590/1980-549720260054)
Supplement: Supplementary file 1 [file 1980-5497-rbepid-29-e260054-Suppl01.docx]

EI: energy intake; TEE: total energy expenditure.

**Supplementary Figure 1. Bland–Altman plots for agreement between dietary estimates and recovery biomarkers.**

IE: ingestão energética; GET: gasto energético total.

**Figura Suplementar 1.** Gráficos de Bland-Altman referentes à concordância entre estimativas dietéticas e biomarcadores de recuperação.
