# Supplementary material for: Validation of the Brasil-Nutri software for 24-hour dietary recall using recovery biomarkers
Source: Rev Bras Epidemiol. 2026 Oct 19;29:e260054. doi: 10.1590/1980-549720260054 (PMC13679881; doi:10.1590/1980-549720260054)
Supplement: Translation

# Translation: Validação do software Brasil-Nutri para recordatórios alimentares de 24 horas utilizando biomarcadores de recuperação

**DOI:** 10.1590/1980-549720260054.2

**Authors:** Jéssica Levy, Letícia Lopes Takarabe, Eduardo De Carli, Sandra Patricia Crispim, Rosângela Alves Pereira, Edna Massae Yokoo, Karina Pfrimer, Daniela Dalpubel Campanari, Meire Gallo Rosenburg, Paulo Lotufo, Isabela Bensenor, Roseli Sichieri, Jeanne Hubertha Maria de Vries, Dirce Maria Lobo Marchioni

**Affiliations:** I Universidade de São Paulo, Faculdade de Saúde Pública, Departamento de Nutrição – São Paulo (SP), Brasil.; II Universidade Federal do Paraná, Departamento de Nutrição – Curitiba (PR), Brasil.; III Universidade Federal do Rio de Janeiro, Departamento de Nutrição Social e Aplicada – Rio de Janeiro (RJ), Brasil.; IV Universidade Federal Fluminense, Instituto de Saúde Coletiva – Niterói (RJ), Brasil.; V Universidade de Ribeirão Preto, Departamento de Nutrição – Ribeirão Preto (SP), Brasil.; VI Universidade de São Paulo, Faculdade de Medicina de Ribeirão Preto – Ribeirão Preto (SP), Brasil.; VII Universidade de São Paulo, Hospital Universitário, Centro de Pesquisa Clínica e Epidemiológica – São Paulo (SP), Brasil.; VIII Universidade Estadual do Rio de Janeiro, Instituto de Medicina Social, Departamento de Epidemiologia – Rio de Janeiro (RJ), Brasil.; IX Wageningen University & Research, Human Nutrition & Health – Wageningen, The Netherlands.

**Keywords:** Software, Inquéritos sobre dietas, Gasto energético, Biomarcador, Estudo de validação

## Abstract

**Objetivo:**

Avaliar erros de medida em recordatórios alimentares de 24 horas duplicados, guiados pelo software Brasil-Nutri, utilizando biomarcadores de recuperação para energia, proteína, sódio e potássio em uma subamostra de participantes do Estudo Longitudinal de Saúde do Adulto (ELSA-Brasil).

**Métodos:**

Homens e mulheres aparentemente saudáveis e com peso corporal estável foram incluídos no estudo. Dois recordatórios alimentares de 24 horas não consecutivos, guiados pelo software Brasil-Nutri, foram comparados com biomarcadores de recuperação de proteína, potássio e sódio obtidos a partir de duas amostras de urina de 24 horas. O gasto energético total foi avaliado por meio do método da água duplamente marcada.

**Resultados:**

Oitenta indivíduos, com idade entre 43 e 72 anos, participaram do estudo. A ingestão energética autorreferida (2.146 kcal/dia) foi inferior ao gasto energético total estimado pelo método da água duplamente marcada (2.526 kcal/dia), com diferença média de 380 kcal/dia. A ingestão autorreferida de proteína (94,9 g/dia) foi superior à estimada pelo biomarcador (87,6 g/dia). Para o sódio, a ingestão média autorreferida foi de 2,9 g/dia em comparação com 4,7 g/dia estimados pelo biomarcador, enquanto a ingestão de potássio foi de 2,9 g/dia *versus* 3,0 g/dia, respectivamente.

**Conclusão:**

O software Brasil-Nutri apresentou desempenho aceitável para estimar a ingestão média populacional de alguns nutrientes; entretanto, erros de medida importantes foram observados, particularmente para a ingestão de sódio. Além disso, as correlações com os biomarcadores de recuperação foram baixas a moderadas, indicando limitações na concordância em nível individual.

## INTRODUÇÃO

A ingestão alimentar é comumente avaliada utilizando-se métodos dietéticos autorrelatados que estão sujeitos a erros aleatórios e sistemáticos^1^. Compreender esses erros é essencial para interpretar ou corrigir os resultados de estudos em epidemiologia nutricional. Para avaliar a validade dos métodos de avaliação dietética, suas estimativas são comparadas com aquelas obtidas a partir de métodos de referência com erros de medição independentes^2,3^. Os avanços nas tecnologias de avaliação dietética reduziram o tempo e os custos necessários para a coleta, codificação e análise de dados. Contudo, a validação desses métodos adaptados também é necessária^4-9^.

O Brasil-Nutri é um sistema informatizado de recordatório alimentar de 24 horas desenvolvido para apoiar inquéritos dietéticos nacionais no Brasil, incluindo o Inquérito Nacional de Alimentação^10-12^. O software inclui uma extensa base de dados de composição de alimentos, fundamentada em inquéritos dietéticos e estudos epidemiológicos nacionais anteriores^13^.

Os biomarcadores de recuperação fornecem medidas objetivas de ingestão alimentar e são amplamente utilizados em estudos de validação porque os seus erros de medição são, em sua maioria, independentes dos métodos autorreferidos^1^. Esses biomarcadores incluem excreções de nitrogênio, sódio e potássio obtidas de amostras de urina de 24 horas, que podem ser usadas para estimar a ingestão de proteínas e minerais na alimentação^14^.

No presente estudo, nosso objetivo foi avaliar erros de medida em recordatórios alimentares de 24 horas duplicados, guiados pelo software Brasil-Nutri, em comparação com biomarcadores de recuperação em uma subamostra de participantes do Estudo Longitudinal de Saúde do Adulto (ELSA-Brasil). A ingestão energética foi avaliada pelo método da água duplamente marcada (ADM), enquanto que a ingestão de proteína, sódio e potássio foi avaliada por meio de biomarcadores urinários de recuperação. O desempenho foi avaliado tanto em nível populacional como individual.

## MÉTODOS

### Amostra do estudo

O ELSA-Brasil é uma investigação de coorte multicêntrica com 15.105 participantes, de ambos os sexos, trabalhadores ativos e aposentados de seis estados brasileiros, com o objetivo principal de examinar a incidência e os fatores de risco para doenças crônicas não transmissíveis^15^. O presente estudo foi realizado no centro de pesquisa ELSA-Brasil em São Paulo, localizado no Centro de Pesquisa Clínica e Epidemiológica do Hospital Universitário da Universidade de São Paulo (CPCE/HU/USP).

Entre agosto de 2018 e dezembro de 2019, foram recrutados 101 adultos e idosos de ambos os sexos. A seleção dos participantes e as exclusões realizadas estão descritas na Figura 1. Para validar as estimativas de ingestão de proteína, sódio e potássio, obtidas por meio do software Brasil-Nutri, 18 indivíduos foram excluídos devido a dados faltantes com relação à avaliação dietética e/ou coleta incompleta de urina de 24 horas. Além disso, três participantes sem informação quanto ao método ADM foram excluídos. Assim, a amostra final da análise foi composta por 80 participantes.

**Figura 1:** Fluxograma de recrutamento voluntário.

Todos os participantes receberam instruções detalhadas quanto ao protocolo de coleta e assinaram um Termo de Consentimento Livre e Esclarecido, indicando sua intenção de participar no estudo. O estudo foi aprovado pelo Comitê de Ética em Pesquisa do Hospital Universitário da Universidade de São Paulo.

### Recordatórios alimentares de 24 horas guiados pelo software Brasil-Nutri

Dois recordatórios alimentares de 24 horas (24hR) não consecutivos, com intervalo de três a quatro semanas entre as avaliações, foram coletados por meio do software Brasil-Nutri. O protocolo do estudo foi concebido para que a primeira entrevista do 24hR fosse realizada presencialmente no centro de estudo e a segunda, por telefone. No entanto, para os participantes que tiveram dificuldade em completar a entrevista por telefone ou que apresentaram limitações relacionadas ao contato remoto, a segunda entrevista também foi realizada presencialmente para minimizar as perdas de seguimento. A estratégia de entrevista por telefone foi adotada visando avaliar a viabilidade operacional da avaliação dietética remota, semelhante aos procedimentos utilizados em grandes estudos epidemiológicos. Além disso, todos os participantes realizaram uma entrevista inicial presencialmente com apoio visual para estimativa do tamanho das porções e medidas domésticas.

A entrevista do 24hR guiada pelo software Brasil-Nutri começa com questões sobre o uso habitual de sal e açúcar ou adoçantes, suplementos nutricionais e adesão a padrões de dieta restritivos ou terapias nutricionais específicas. Os entrevistados foram solicitados a mencionar todos os alimentos e bebidas consumidos nas últimas 24 horas, o local e o tempo de consumo, bem como detalhes sobre cada alimento e bebida, incluindo o método de preparação, o tipo de unidade e a quantidade de consumo em medições domésticas ou unidades padrão, ilustrados por imagens em uma tela de computador^16^. A estimativa de nutrientes foi baseada na Tabela Brasileira de Composição de Alimentos (TBCA/USP)^17-19^. Os valores de sódio disponíveis na base de dados de composição de alimentos incluem o sódio intrínseco, o sódio adicionado durante o processamento de alimentos e o sódio estimado com base no preparo de alimentos.

### Dados antropométricos e de estilo de vida

Cada participante preencheu um questionário sobre caraterísticas socioeconômicas e fatores de estilo de vida. A atividade física foi classificada de acordo com o Questionário Internacional de Atividade Física (*International Physical Activity Questionnaire* – IPAQ) como leve, moderada ou vigorosa^20^. Devido ao pequeno número de participantes na categoria "vigorosa", níveis moderados e vigorosos foram combinados. A classe social foi categorizada de acordo com os critérios de classificação econômica da Associação Brasileira de Empresas de Pesquisa, com ≥45 pontos para a classe alta; ≥29 e ≤44 pontos para a classe média; e ≤28 pontos para a classe baixa^21^.

As medidas antropométricas foram realizadas de forma duplicada, com os participantes vestindo roupas leves, sem sapatos e adornos e com bolsos vazios. A altura, o peso e a circunferência da cintura foram medidos com aproximação de 0,1 cm, 0,1 kg e 0,1 cm, respectivamente, com equipamento calibrado no início e no final do estudo. Foram utilizadas medidas médias de peso e altura para calcular o Índice de Massa Corporal (IMC [kg/m^2^]) e classificar o estado nutricional dos indivíduos, seguindo os critérios da Organização Mundial da Saúde^22^ para adultos e os critérios de Lipschitz^23^ para idosos. Os participantes foram classificados como: 1) Subpeso, com IMC<18,5 kg/m^2^ para adultos e IMC<22 kg/m^2^ para idosos; 2) peso normal, considerando IMC 18,5–24,9 kg/m² para adultos e IMC<27 kg/m^2^ para idosos; e 3) sobrepeso, com IMC≥25 kg/m^2^ para adultos e IMC≥27 kg/m^2^ para idosos. No entanto, como apenas um participante estava abaixo do peso, a categoria "subpeso" foi combinada com o grupo de peso normal.

### Biomarcadores de recuperação

Duas amostras de urina de 24 horas (24hU) foram coletadas de cada participante como parte do protocolo do estudo. A primeira amostra de 24hU foi coletada no mesmo dia da primeira avaliação do 24hR. A segunda amostra de 24hU foi coletada após a conclusão das duas avaliações do 24hR, durante um período de avaliação subsequente. O intervalo médio entre as coletas de urina foi de seis meses (mínimo de um e máximo de 14 meses). Para as análises, utilizou-se a média das duas coletas de urina como medida de biomarcador de referência visando reduzir a variância intrapessoal e melhorar a estimativa da ingestão habitual.

As coletas de 24hU que tinham um intervalo de coleta menor que 22 horas ou maior que 26 horas foram consideradas inválidas. O tempo total de coleta de urina foi calculado como o intervalo entre os tempos de início e fim da coleta. Coletas com um volume de 24hU inferior a 500 mL ou com excreção total de creatinina fora da faixa esperada, <14,4 ou >33,6 mg/kg para homens e <10,8 ou >25,2 mg/kg para mulheres, conforme sugerido por Pereira et al.^24^, também foram consideradas inválidas.

A determinação da ureia na urina foi realizada por um método cinético (urease e a glutamato desidrogenase), realizado pelo analisador automático Cobas c501^®^ (Roche^®^). Os valores da excreção total de ureia em 24 horas foram convertidos em nitrogênio (g/dia), considerando que a ureia é uma porção constante/estável (85%) do nitrogênio total da urina^25,26^. Em seguida, a ingestão de proteínas foi calculada utilizando a seguinte fórmula: ingestão de proteínas = 6,25 * (nitrogênio urinário [g/dia] + 2), onde + 2 representa perdas de nitrogênio extra-renal (fezes e pele) em g/dia^27^. As concentrações de potássio urinário foram medidas utilizando a técnica de eletrodo íon-seletivo. A ingestão de potássio foi calculada dividindo-se a excreção de potássio urinário por 0,77. As concentrações de sódio urinário foram medidas utilizando a técnica de eletrodo íon-seletivo. A ingestão de sódio foi calculada dividindo-se a excreção de sódio urinário por 0,86, de acordo com Freedman et al.^28^.

O gasto energético total (GET) durante um período de duas semanas foi avaliado utilizando o método da água duplamente marcada (ADM) em 101 participantes. O protocolo de ADM foi repetido em 26 participantes para avaliar a reprodutibilidade e melhorar a precisão das estimativas de GET. As doses foram preparadas considerando a razão de 2 g de 10% ^18^O e 0,11 g de óxido de deutério (^2^H_2_O) de 99,8% por quilograma de água corporal total estimada^29^. Uma amostra basal de urina foi coletada antes da ingestão da dose de ADM pelos participantes. Após a ingestão, uma amostra foi coletada todos os dias durante 14 dias, preferencialmente no mesmo horário nas casas dos participantes. Nos 7º e 14º dias de coleta, as amostras da semana foram entregues ao laboratório, e as amostras do dia foram coletadas no momento da entrevista no centro de pesquisa. No entanto, apenas amostras basais e amostras dos dias 1, 2, 3, 7, 8, 13 e 14 foram analisadas. Todas as amostras foram refrigeradas até serem entregues para processamento. O enriquecimento isotópico de ^2^H e ^18^O em amostras de urina foi analisado em triplicata utilizando espectrometria de massa de razões isotópicas (IRMS-GLS: ANCA 20-20, Europe Scientific, Reino Unido e ANCA 22-20, Sercon, Reiuno Unido) no Laboratório de Espectrometria de Massa de Razões Isotópicas da Faculdade de Medicina de Ribeirão Preto, Universidade de São Paulo, Brasil. Os valores da taxa de decaimento entre ²H e ^18^O variaram de 1,01 a 1,07. O peso corporal foi monitorado durante todo o período de medição da ADM para garantir que os participantes não estavam perdendo ou ganhando peso. Os participantes foram considerados estáveis quando a diferença de peso entre os dias 1 e 14 foi de <1 kg.

### Análise estatística

Os dados referentes às caraterísticas socioeconômicas, antropométricas e de estilo de vida foram descritos por frequências, e as associações entre as variáveis categóricas foram testadas por meio do teste χ² ou pelo teste exato de Fisher. Os valores das variáveis dietéticas e dos biomarcadores foram apresentados em forma bruta. As diferenças (ingestão alimentar menos biomarcador), razões (ingestão alimentar/biomarcador) e viés relativo {[(ln ingestão alimentar/ln biomarcadores) × 100] − 100}, representando o percentual de subnotificação em nível populacional, foram estimados^28^. A concordância entre os métodos, tanto em nível populacional como em nível individual, global e estratificada por sexo, foi avaliada utilizando os gráficos de Bland-Altman^30^. Para as demais análises, os valores obtidos em dois dias não consecutivos foram ajustados para a distribuição da ingestão por variância intrapessoal, utilizando o software *Multiple Source Method* (MSM)^31^. Os coeficientes de correlação entre as variáveis dietéticas e os seus respetivos biomarcadores para avaliar se o método foi capaz de classificar os indivíduos de acordo com a sua ingestão foram calculados utilizando o coeficiente de correlação de Pearson, e as transformações Z de Fisher foram usadas para calcular os intervalos de confiança de 95% (IC 95%) dos coeficientes de correlação. Em todas as análises, um nível de significância de 5% foi considerado, e o processamento estatístico dos dados foi realizado utilizando o software Stata^®^ (versão 14.0).

Para interpretar a validade do método de avaliação dietética, diferentes aspetos de desempenho foram considerados. A concordância em nível populacional foi avaliada comparando as estimativas médias de ingestão alimentar com os biomarcadores de recuperação por meio de diferenças, razões e viés relativo. A capacidade de classificar os indivíduos de acordo com a ingestão alimentar foi avaliada por meio de coeficientes de correlação entre estimativas dietéticas e biomarcadores, com correlações classificadas como baixas (<0,30), moderadas (0,30–0,50) ou altas (>0,50). A concordância em nível individual foi adicionalmente avaliada de acordo com os gráficos de Bland–Altman e limites de concordância.

#### Declaração de disponibilidade de dados:

Todo o conjunto de dados que dá suporte aos resultados deste estudo está disponível mediante solicitação e aprovação pelo presidente da comissão diretiva para o estudo de validação do software Brasil-Nutri do Estudo Longitudinal de Saúde do Adulto (ELSA-Brasil).

## RESULTADOS

Entre os 80 participantes do estudo, 43 eram mulheres e 37 eram homens. A maioria (86,3%) tinha entre 43 e 59 anos. Mais da metade tinha Ensino Superior completo ou diploma de pós-graduação (56,2%), 62,5% pertenciam à classe social média, 55,0% reportaram atividade física leve e 63,8% tiveram seu peso classificado como sobrepeso (Tabela 1).

**Tabela 1:** Características socioeconômicas e de estilo de vida de uma subamostra do Estudo Longitudinal de Saúde do Adulto utilizada para a validação do software Brasil-Nutri.

		Total (n=80)		Homens (n=37)		Mulheres (n=43)		p*
		n	%	n	%	n	%	
Faixa etária								
	Adultos	69	86,3	32	86,5	37	86,1	0,955
	Idosos	11	13,7	5	13,5	6	13,9	
Escolaridade								
	Ensino Médio ou menos	35	43,8	19	51,4	16	37,2	0,204
	Ensino Superior/Pós-graduação	45	56,2	18	48,6	27	62,8	
Classe social								
	Alta	17	21,2	7	18,9	10	23,3	0,787
	Média	50	62,5	23	62,2	27	62,8	
	Baixa	13	16,2	7	18,9	6	13,9	
Índice de Massa Corporal								
	Peso normal	29	36,2	9	24,3	20	46,5	0,040
	Sobrepeso	51	63,8	28	75,7	23	53,5	
Atividade física								
	Leve	44	55,0	19	51,4	25	58,1	0,543
	Moderada a vigorosa	36	45,0	18	48,6	18	41,9	

Os valores apresentados são a soma (frequência);

* p-valor relativo à comparação entre os sexos de acordo com o teste χ².

Adultos: 43 a 59 anos; Idosos: 60 anos ou mais. Classe social de acordo com os critérios da Associação Brasileira de Empresas de Pesquisa (ABEP): a classe alta é maior ou igual a 45 pontos; a classe média, maior ou igual a 29 e menor ou igual a 44 pontos; a classe baixa, menor ou igual a 28 pontos. Índice de Massa Corporal: o peso normal é inferior a 25 kg/m^2^ para adultos e inferior a 27 kg/m^2^ para idosos; o sobrepeso é maior ou igual a 25 kg/m^2^ para adultos e maior ou igual a 27 kg/m^2^ para idosos. Atividade física leve: indivíduos que não realizaram nenhuma atividade física por pelo menos 10 minutos contínuos durante a semana ou que realizaram atividade física, mas insuficientemente para serem classificados como ativos por não atenderem às recomendações quanto à frequência (maior ou igual a cinco dias por semana) ou duração (maior ou igual a 150 minutos por semana). Atividade física moderada a vigorosa: indivíduos que atenderam às recomendações de maior ou igual a três dias por semana e maior ou igual a 20 minutos por sessão de atividade vigorosa; maior ou igual a cinco dias por semana e maior ou igual a 30 minutos por sessão de atividade moderada ou caminhada; ou maior ou igual a cinco dias por semana e maior ou igual a 150 minutos em qualquer atividade adicionada.

Na Tabela 2, apresentamos as estimativas de ingestão alimentar para energia, proteína, sódio e potássio e seus respectivos biomarcadores de recuperação e as correlações entre os dois métodos. A média de ingestão energética autorreferida (2,146.1 kcal/dia) foi menor que o GET estimado pelo método ADM (2,526.5 kcal/dia), resultando em uma diferença média de 380,3 kcal/dia. Considerando o viés relativo, houve subestimação da ingestão energética na amostra total (-1,8%) bem como entre homens (-1,6%) e mulheres (-2,0%).

**Tabela 2:** Ingestão energética e biomarcador, proteína calculada a partir de nitrogênio, sódio e potássio, com base em dois dias de recordatórios de 24 horas e duas coletas de urina de 24 horas ou método de água duplamente marcada no estudo de validação Brasil-Nutri.

Variáveis		Total (n=80)		Homens (n=37)		Mulheres (n=43)	
		Média	IC 95%	Média	IC 95%	Média	IC 95%
Energia							
	Ingestão (kcal/dia)	2.146,1	(1.995,7 a 2.296,5)	2.413,9	(2.179 a 2.48,9)	1.915,6	(1.742,7 a 2.088,6)
	Biomarcador (kcal/dia)	2.526,5	(2.316,1 a 2.736,9)	2.834,9	(2.489,5 a 3.180,4)	2.261,1	(2.021,8 a 2.500,4)
	Diferença (kcal/dia)	-380,3	(-617,8 a -142,9)	-421,0	(-809,5 a -32,56)	-345,4	(-649,2 a -41,6)
	Razão	0,9	(0,8 a 1,0)	0,9	(0,8 a 1,1)	0,9	(0,8 a 1,0)
	Viés relativo (%)	-1,8	(-3,0 a -0,6)	-1,6	(-3,5 a 0,3)	-2,0	(-3,7 a -0,4)
	Correlação bruta	0,15	(-0,07 a 0,36)	0,15	(-0,19 a 0,45)	-0,02	(-0,32 a 0,28)
Proteína							
	Ingestão (g/dia)	94,9	(87,2 a 102,7)	111,5	(100,4 a 122,7)	80,6	(71,5 a 89,8)
	Biomarcador (g/dia)	87,6	(83,2 a 92,1)	98,1	(91,6 a 104,6)	78,7	(73,8 a 83,5)
	Diferença (g/dia)	7,3	(0,7 a 13,9)	13,5	(2,6 a 24,3)	1,9	(-6,1 a 10,1)
	Razão	1,1	(1,0 a 1,1)	1,2	(1,0 a 1,3)	1,0	(0,9 a 1,1)
	Viés relativo (%)	0,8	(-0,7 a 2,42)	2,3	(0,1 a 4,5)	-0,4	(-2,7 a 1,8)
	Correlação bruta	0,6	(0,39 a 0,69)	0,4	(0,10 a 0,65)	0,5	(0,23 a 0,69)
	Correlação deatenuada^1^	0,6	(0,46 a 0,74)	0,3	(0,02 a 0,59)	0,4	(0,06 a 0,59)
Sódio							
	Ingestão (g/dia)	2,9	(2,7 a 3,3)	3,5	(3,0 a 3,9)	2,6	(2,3 a 2,9)
	Biomarcador (g/dia)	4,7	(4,4 a 5,0)	5,6	(5,1 a 6,1)	3,9	(3,6 a 4,2)
	Diferença (g/dia)	-1,7	(-2,1 a -1,3)	-2,1	(-2,8 a -1,4)	-1,4	(-1,7 a -0,9)
	Razão	0,7	(0,6 a 0,8)	0,7	(0,6 a 0,8)	0,7	(0,6 a 0,8)
	Viés relativo (%)	-30,7	(-37,1 a -24,3)	-27,7	(-37,5 a -18,0)	-33,2	(-42,1 a -24,4)
	Correlação bruta	0,37	(0,16 a 0,55)	0,12	(-0,21 a 0,43)	0,27	(-0,03 a 0,53)
	Correlação deatenuada^1^	0,52	(0,33 a 0,66)	0,02	(-0,31 a 0,34)	0,11	(-0,19 a 0,39)
Potássio							
	Ingestão (g/dia)	2,9	(2,7 a 3,1)	3,2	(2,9 a 3,5)	2,6	(2,3 a 2,9)
	Biomarcador (g/dia)	3,0	(2,9 a 3,2)	3,2	(2,9 a 3,5)	2,9	(2,6 a 3,1)
	Diferença (g/dia)	-0,2	(-0,4 a 0,1)	-0,0	(-0,3 a 0,3)	-0,3	(-0,6 a 0,1)
	Razão	0,9	(0,9 a 1,1)	1,0	(0,9 a 1,1)	0,9	(0,8 a 1,1)
	Viés relativo (%)	-3,7	(-11,9 a 4,6)	-0,1	(-10,6 a 10,3)	-6,7	(-19,6 a 6,1)
	Correlação bruta	0,28	(0,06 a 0,47)	0,53	(0,24 a 0,73)	0,01	(-0,29 a 0,31)
	Correlação deatenuada^1^	0,33	(0,12 a 0,51)	0,34	(0,02 a 0,60)	0,19	(-0,11 a 0,47)

Os valores apresentados são médias e intervalos de confiança de 95% (IC 95%); Diferença: ingestão alimentar – biomarcador; Razão: ingestão alimentar dividido por biomarcador; Viés relativo: {[ln ingestão alimentar / ln biomarcador) x 100] – 100]}.

* As medidas de ingestão alimentar e biomarcadores foram obtidas a partir de recordatórios de 24 horas e de coletas de urina de 24 horas, respectivamente.

1 Correlação com a remoção da variância intrapessoal por meio do método *Multiple Source Method* (MSM).

A média de ingestão de proteína autorreferida (94,9 g/dia) foi maior do que a estimativa do biomarcador (87,6 g/dia), correspondendo a uma diferença média de 7,3 g/dia. Entre os homens, a ingestão de proteína autorreferida foi de 111,5 g/dia comparado com 98,1 g/dia estimado pelo biomarcador, enquanto que entre as mulheres os valores correspondentes foram de 80,6 g/dia e 78,7 g/dia. O coeficiente de correlação entre a ingestão de proteína e o seu biomarcador foi de 0,60 (IC 95% 0,39–0,69), mantendo-se inalterado após ajuste para a variância intrapessoal (r=0,60; IC 95% 0,46–0,74). Para o sódio, a ingestão média autorreferida foi de 2,9 g/dia, enquanto a estimativa do biomarcador foi de 4,7 g/dia, resultando em uma diferença média de 1,7 g/dia. Para o potássio, a ingestão média autorreferida foi de 2,9 g/dia, comparado com 3,0 g/dia estimado pelo biomarcador, correspondendo a uma diferença média de 0,2 g/dia.

Na Tabela 3, apresentamos as diferenças médias entre as estimativas de ingestão alimentar e os biomarcadores de recuperação segundo as caraterísticas socioeconômicas, antropométricas e de estilo de vida. Não observamos diferenças estatisticamente significativas entre os grupos avaliados. No entanto, verificamos a subestimação para a ingestão energética, de sódio e potássio na maioria dos subgrupos. Para a ingestão energética, observamos maior subestimação entre idosos, participantes com sobrepeso e aqueles que relataram atividade física moderada a vigorosa. Identificamos um padrão distinto entre os grupos socioeconômicos, com sobrenotificação entre os participantes da classe social mais baixa e subnotificação entre os participantes das classes média e alta. Para o sódio e o potássio, as diferenças entre as estimativas dietéticas e os biomarcadores foram relativamente consistentes entre subgrupos, embora maiores discrepâncias entre os idosos tenham sido observadas. Por outro lado, o consumo de proteína tendeu a ser superestimado entre os adultos, participantes da classe social baixa, indivíduos com sobrepeso e aqueles que relataram atividade física leve.

**Tabela 3:** Médias e intervalos de confiança de 95% da diferença entre estimativas dietéticas e biomarcadores para energia, proteína, sódio e potássio no estudo de validação do software Brasil-Nutri.

Características		Diferença de energia (kcal)		Diferença de proteína (g)		Diferença de sódio (g)		Diferença de (g) potássio (g)	
Faixa etária									
	Adultos	-321,3	(-586,9 a -55,8)	7,5	(0,0 a 14,9)	-1,7	(-2,0 a -1,2)	-0,1	(-0,3 a 0,2)
	Idosos	-750,7	(-1.229,1 a -272,4)	6,1	(-8,4 a 20,7)	-2,2	(-3,2 a -0,8)	-0,6	(-1,4 a 0,2)
Escolaridade									
	Ensino Médio ou menos	-488,4	(-882,4 a -4,3)	6,9	(-2,8 a 16,7)	-1,9	(-2,6 a -1,2)	-0,2	(-0,5 a 0,2)
	Ensino Superior/Pós-graduação	-327,5	(-596,7 a -58,3)	7,6	(-1,8 a 16,9)	-1,6	(-2,0 a -1,1)	-0,1	(-0,5 a 0,2)
Classe social									
	Alta	-493,8	(-1.004,6 a 16,8)	0,8	(-5,6 a 7,2)	-1,5	(-2,2 a -0,9)	-0,6	(-1,0 a -0,1)
	Média	-449,6	(-760,2 a -139,1)	8,8	(-1,1 a 18,7)	-1,7	(-2,2 a -1,2)	-0,1	(-0,4 a 0,2)
	Baixa	34,4	(-592,1 a 661,0)	9,9	(-5,0 a 24,8)	-1,8	(-3,0 a -0,6)	0,1	(-0,5 a 0,7)
Índice de Massa Corporal									
	Peso normal	-185,0	(-429,4 a 59,3)	1,9	(-10,7 a 14,7)	-1,5	(-1,9 a -0,9)	-0,5	(-0,9 a 0)
	Sobrepeso	-491,5	(-839,6 a -143,3)	10,3	(2,6 a 17,9)	-1,8	(-2,4 a -1,3)	0,0	(-0,2 a 0,3)
Atividade física									
	Leve	-276,6	(-547,2 a -6,0)	8,6	(-0,9 a 18,1)	-1,6	(-2,1 a -1,1)	-0,2	(-0,5 a 0,2)
	Moderada a vigorosa	-507,2	(-930,5 a -83,9)	5,6	(-3,9 a 15,1)	-1,8	(-2,4 a -1,3)	-0,1	(-0,5 a 0,2)

* Os valores negativos indicam subestimação.

Adultos: 43 a 59 anos; Idosos: 60 anos ou mais. Classe social de acordo com os critérios da Associação Brasileira de Empresas de Pesquisa (ABEP): a classe alta é maior ou igual a 45 pontos; a classe média, maior ou igual a 29 e menor ou igual a 44 pontos; a classe baixa, menor ou igual a 28 pontos. Índice de Massa Corporal: o peso normal é inferior a 25 kg/m^2^ para adultos e inferior a 27 kg/m^2^ para idosos; o sobrepeso é maior ou igual a 25 kg/m^2^ para adultos e maior ou igual a 27 kg/m^2^ para idosos. Atividade física leve: indivíduos que não realizaram nenhuma atividade física por pelo menos 10 minutos contínuos durante a semana ou que realizaram atividade física, mas insuficientemente para serem classificados como ativos por não atenderem às recomendações quanto à frequência (maior ou igual a cinco dias por semana) ou duração (maior ou igual a 150 minutos por semana). Atividade física moderada a vigorosa: indivíduos que atenderam às recomendações de maior ou igual a três dias por semana e maior ou igual a 20 minutos por sessão de atividade vigorosa; maior ou igual a cinco dias por semana e maior ou igual a 30 minutos por sessão de atividade moderada ou caminhada; ou maior ou igual a cinco dias por semana e maior ou igual a 150 minutos em qualquer atividade adicionada.

De acordo com as análises de concordância baseadas nos gráficos de Bland-Altman, houve uma variância substancial entre as estimativas dietéticas e os biomarcadores de recuperação em nível individual, particularmente para a ingestão energética e de sódio (Figura Suplementar 1). O potássio apresentou limites de concordância comparativamente menores, enquanto o sódio apresentou a maior diferença entre os métodos.

## DISCUSSÃO

Neste estudo, avaliamos os erros de medição em recordatórios alimentares de 24 horas guiados pelo software Brasil-Nutri utilizando biomarcadores de recuperação em participantes do estudo ELSA-Brasil. Em nível populacional, o desempenho do método variou de acordo com o nutriente avaliado, com superestimação para proteína e subestimação para sódio e potássio quando comparados com biomarcadores de recuperação. Além disso, observamos correlações baixas a moderadas entre estimativas dietéticas e biomarcadores, particularmente para a ingestão energética, indicando concordância limitada em nível individual. Tais achados sugerem que, embora o software possa fornecer estimativas razoáveis para alguns nutrientes em nível populacional, erros de medição significantes permanecem e devem ser cuidadosamente considerados ao interpretar os resultados.

Um número crescente de pesquisadores tem proposto inovações tecnológicas para a coleta de 24hR, incluindo o uso, por exemplo, de aplicativos de *smartphones* e imagens digitais, visando superar desafios logísticos em estudos epidemiológicos^5-9,32,33^. Autores de estudos anteriores de validação de ferramentas online de avaliação dietética, como o Oxford WebQ e MyFood24, também relataram erros de medição quando comparados com biomarcadores de recuperação, incluindo a superestimação de alguns nutrientes^32,33^. Autores de outras pesquisas, nas quais estimativas de proteína, sódio e potássio obtidas pelo 24hR foram comparadas com biomarcadores urinários, relataram tendência a subestimar o consumo dos três nutrientes^34-36^. No presente estudo, identificamos uma superestimação para proteína (94,9 g *versus* 87,6 g) e subestimação para sódio (2,9 g *versus* 4,7 g) e potássio (2,9 g *versus* 3,0 g). Em contraste com a subestimação usualmente descrita para os métodos de avaliação da ingestão alimentar, a ingestão de proteínas foi levemente superestimada no presente estudo. Uma possível explicação é que os alimentos ricos em proteínas são geralmente mais fáceis de identificar e quantificar em recordatórios alimentares do que nutrientes como o sódio, que dependem fortemente das práticas de preparação de alimentos e do uso discricionário de sal. No mais, o nível de escolaridade relativamente elevado da população do estudo pode ter contribuído para uma informação mais precisa das principais refeições e dos alimentos que contêm proteínas.

Verificamos diferenças comparativamente menores e limites de concordância mais estreitos no potássio do que no sódio. Esse achado pode estar relacionado com o fato de a ingestão de potássio ser amplamente derivada de alimentos minimamente processados, como frutas, vegetais e legumes, que são geralmente mais fáceis de relatar e menos influenciados por práticas de preparação discricionária.

Autores de um estudo norte-americano, no qual biomarcadores de recuperação foram utilizados como método de referência e apenas mulheres foram incluídas, relataram um coeficiente de correlação deatenuado de 0,54 para ingestão de proteína ajustado pela energia, avaliado usando o *Automated Self-Administered 24-Hour Dietary Recall* (ASA24)^37^, que é comparável à correlação observada no presente estudo (r=0,60)^37^. Os coeficientes de correlação observados no nosso estudo foram comparáveis aos relatados em estudos anteriores de validação utilizando biomarcadores urinários^38^. Para o potássio, as correlações deatenuadas foram de 0,34 para os homens e 0,19 para as mulheres. Para o sódio, os valores correspondentes foram de 0,02 e 0,11, respectivamente. Embora de baixa a moderada, essas correlações indicam que os recordatórios alimentares foram capazes de classificar os indivíduos de acordo com a ingestão, o que é uma importante caraterística para estudos epidemiológicos.

A subestimação significativa observada para a ingestão de sódio representa um dos principais achados deste estudo e destaca uma importante limitação do método. Estimar a ingestão de sódio por meio de avaliação dietética autorreferida continua a ser um dos maiores desafios na avaliação dietética devido às dificuldades em quantificar o sal discricionário adicionado durante a preparação e o consumo de alimentos, bem como à variabilidade do teor de sódio nos alimentos processados. Além disso, a ingestão de sódio apresenta uma variância intrapessoal considerável, o que pode reduzir ainda mais a concordância entre os recordatórios alimentares e os biomarcadores de recuperação. O peso corporal tem sido consistentemente identificado como um preditor de viés de medida em métodos de avaliação dietética autorreferida, com maior grau de subnotificação, especialmente em relação à energia, associado ao sobrepeso e à obesidade^39-45^. Mercado et al.^38^ descreveram uma maior subnotificação da ingestão de sódio em 24hR, identificada pelo método tradicional entre indivíduos com sobrepeso e obesidade. No presente estudo, as estimativas de sódio obtidas pelo 24hR em comparação com o seu biomarcador foram subestimadas em 1,7 g para a população total e em 2,1 g e 1,4 g para homens e mulheres, respectivamente. No mais, a subestimação foi maior entre os participantes com sobrepeso em comparação com os participantes com peso normal especificamente para esse nutriente (1,8 g *versus* 1,5 g). No entanto, vale destacar que indivíduos com obesidade foram excluídos do presente estudo como uma estratégia metodológica deliberada para minimizar os fatores anteriormente associados à subnotificação sistemática em estudos de avaliação dietética. Autores de estudos anteriores que comparavam recordatórios alimentares com biomarcadores urinários também demonstraram baixa concordância e subestimação significativa para o consumo de sódio em nível individual^46-49^.

As comparações com estudos envolvendo populações obesas devem, portanto, ser interpretadas com cautela; foram incluídas aqui principalmente para contextualizar padrões de erros de subnotificação dietética descritos na literatura. A aplicabilidade dos nossos achados aos indivíduos com obesidade permanece incerta e deve ser investigada em futuros estudos de validação que incluam populações mais heterogêneas.

Outro aspeto importante é que estimar a ingestão discricionária de sal permanece inerentemente desafiador em inquéritos dietéticos, mesmo quando questões específicas sobre o sal adicionado são incluídas. No software Brasil-Nutri, questões adicionais relacionadas à preparação de alimentos e tempero, incluindo adição de sal, foram sistematicamente aplicadas a todos os alimentos relatados. No entanto, os participantes podem deixar de relatar o sal adicionado a saladas e alimentos preparados em casa. Tais fatores podem ter contribuído para a subestimação significativa observada para a ingestão de sódio no presente estudo.

Freedman et al. agrupou dados de vários estudos de validação e reportou subnotificação de ingestão energética numa faixa de 6 a 28%, dependendo da população e do sexo do estudo^28^. Em estudos com o ASA 24, a ingestão energética estimada foi menor do que o GET para homens e mulheres^50,51^. No presente estudo, a subnotificação da ingestão energética com dois 24hR por meio do software Brasil-Nutri foi de 1,6% para homens e 2,0% para mulheres. A subestimação observada para a ingestão energética pode ser parcialmente explicada por omissões de alimentos percebidos como pouco saudáveis, dificuldades em lembrar lanches e alimentos consumidos fora de casa e desafios na estimativa do tamanho das porções. Ademais, a ingestão energética é particularmente suscetível a tendências de conveniência social e subnotificação seletiva, mesmo entre indivíduos aparentemente saudáveis.

O estudo tem vários pontos fortes, incluindo o uso de critérios rigorosos de seleção de participantes, projetados para minimizar os fatores associados à subnotificação dietética, como doenças crônicas, uso de medicamentos/suplementos ou condições fisiológicas que possam comprometer a validade de biomarcadores ou a comunicação de consumo; o uso de biomarcadores de recuperação para validação de proteínas, sódio e potássio; e procedimentos rigorosos de controle de qualidade durante a coleta, armazenamento e análise laboratorial de urina. Embora a amostra selecionada limite a validade externa, a inclusão de indivíduos aparentemente saudáveis sem obesidade ou doenças crônicas foi uma estratégia metodológica deliberada para minimizar os fatores associados à subnotificação sistemática já descritos na literatura. Essa abordagem possibilitou a avaliação do desempenho do software em condições mais controladas antes de uma aplicação mais ampla em populações mais heterogêneas. Portanto, os resultados não devem ser extrapolados diretamente para a população brasileira como um todo. Outra limitação do estudo é que a segunda avaliação do biomarcador foi realizada durante um período de avaliação posterior, com um intervalo médio de seis meses entre as coletas de urina. No mais, a estimativa do tamanho das porções durante entrevistas por telefone não teve suporte de imagens digitais de alimentos.

O software Brasil-Nutri demonstrou um desempenho aceitável para estimar a ingestão média em nível populacional de alguns nutrientes; no entanto, erros significativos de medição permanecem, particularmente para a ingestão de sódio. As correlações com os biomarcadores de recuperação foram de baixas a moderadas, sugerindo limitações de concordância em nível individual. Os achados corroboram o uso do método, com cautela, em estudos populacionais. Destacamos a necessidade de maior refinamento, especialmente para a estimativa de sódio.

## Material suplementar

Material suplementar
